# Rapid and Sensitive Detection of miRNA Based on AC Electrokinetic Capacitive Sensing for Point-of-Care Applications

**DOI:** 10.3390/s21123985

**Published:** 2021-06-09

**Authors:** Nan Wan, Yu Jiang, Jiamei Huang, Rania Oueslati, Shigetoshi Eda, Jayne Wu, Xiaogang Lin

**Affiliations:** 1 Key Laboratory of Optoelectronic Technology and System of the Education Ministry of China, Chongqing University, Chongqing 400044, China; wannan@sjtu.edu.cn; 2 Department of Electrical Engineering and Computer Science, The University of Tennessee, Knoxville, TN 37996, USA; yjiang33@vols.utk.edu (Y.J.); jhuang37@vols.utk.edu (J.H.); oueslati.rania91@gmail.com (R.O.); 3 University of Michigan-Shanghai Jiao Tong University Joint Institute, Shanghai Jiao Tong University, Shanghai 200240, China; 4 Department of Forestry, Wildlife and Fisheries, The University of Tennessee Institute of Agriculture, Knoxville, TN 37996, USA; seda@utk.edu

**Keywords:** capacitive sensing, alternating current electrokinetic effects, miRNA sensing, point-of-care diagnostics

## Abstract

A sensitive and efficient method for microRNAs (miRNAs) detection is strongly desired by clinicians and, in recent years, the search for such a method has drawn much attention. There has been significant interest in using miRNA as biomarkers for multiple diseases and conditions in clinical diagnostics. Presently, most miRNA detection methods suffer from drawbacks, e.g., low sensitivity, long assay time, expensive equipment, trained personnel, or unsuitability for point-of-care. New methodologies are needed to overcome these limitations to allow rapid, sensitive, low-cost, easy-to-use, and portable methods for miRNA detection at the point of care. In this work, to overcome these shortcomings, we integrated capacitive sensing and alternating current electrokinetic effects to detect specific miRNA-16b molecules, as a model, with the limit of detection reaching 1.0 femto molar (fM) levels. The specificity of the sensor was verified by testing miRNA-25, which has the same length as miRNA-16b. The sensor we developed demonstrated significant improvements in sensitivity, response time and cost over other miRNA detection methods, and has application potential at point-of-care.

## 1. Introduction

MicroRNAs (miRNAs) are noncoding small RNAs of 18–25 nucleotides that regulate the expression of multiple genes. As the presence of distinct miRNAs indicates specific medical conditions—and because miRNAs can stably exist in various body fluids—miRNAs have been investigated as biomarkers for disease diagnosis [1]. The exosome-associated miRNA has been demonstrated to show potential diagnostic value in Alzheimer’s disease, mild cognitive impairment and lung cancer [2,3]. Extracellular miRNAs not only circulate in the peripheral blood [4,5,6,7], but also widely exist in other body fluids such as saliva, urine, tears, amniotic fluid and breast milk [7,8,9]. For miRNA detection, Northern blotting (NB) is commonly used in molecular biology laboratories; however, it suffers the shortcomings of low sensitivity and long assay time. Reverse transcription quantitative polymerase chain reaction (RT-qPCR), as the gold standard for RNA quantification [10], requires unwieldy and expensive thermal cycling equipment for amplification and quantification, rendering it unsuitable for point-of-care (POC) detection of circulating miRNAs diagnostics. Furthermore, due to the short lengths of miRNAs, RT-PCR of miRNA is rather costly and technically demanding to perform, even compared with regular RT-PCR applications.

To overcome the drawbacks of existing methods for miRNA detection, we have presented herein the development of a rapid, high sensitivity, and easy-to-use biosensor for miRNA POC detection, based on the AC electrokinetic (ACEK)-enhanced capacitive sensing technique. ACEK effects, including the dielectrophoresis (DEP), AC electroosmosis (ACEO), and AC electrothermal (ACET) effects, have been widely studied and applied on biofluid handling and molecule manipulation [11,12]. DEP refers to the particle motion caused by the difference in polarizability between the particles and the fluid [13]. ACEO is induced by the moving charges in the electric double layer (EDL) and induces the motion of fluid [14]. The ACET effect arises from the induced temperature gradient in the fluids [15]. For relatively conductive biofluids, ACET effect dominates ACEO when generating microflows. In the ACEK capacitive sensing method utilized, AC capacitive sensing transduces biomolecular interactions to the change of interfacial capacitance. In the meantime, the AC electrical signal induces the ACET effect, which enriches biomolecules toward the electrode surface for sensing. Thus, rapid and sensitive detection for various biochemical molecules can be realized in a manner amenable to POC applications. In our previous work, we successfully detected progesterone, DNA, RNA, proteins, Bisphenol A and pathogens with the application of ACEK capacitive sensing [16,17,18,19,20].

In this work, miRNA-16b was adopted as a model target to perform a proof-of-concept demonstration. MiRNA-16b was derived from circulating extracellular vesicles and could potentially be used for early identification of the pregnancy status of cattle [21]. To be cost-effective for POC applications, interdigitated electrodes (based on low-cost gold-plated interdigitated printed circuit broad (PCB) electrodes) were adopted as sensors. A single-stranded DNA oligonucleotide probe was functionalized on the electrodes to specifically recognize miRNA-16b. The hybridization of the DNA probe with miRNA-16b resulted in a change at the electrode-electrolyte interface. Microfluidic experiments were conducted to show that the ACET effect dominated over other electrokinetic effects. It was used to accelerate the miRNA-16b target’s travel to the sensor surface and speed up the binding of miRNA-16b with the capture DNA probe—shortening the testing time to 30s. With the combination of DNA probe recognition and ACET-enhanced capacitance sensing, we successfully detected miRNA-16b for bovine pregnancy diagnosis at the femtomolar (fM) level. To test the sensors’ specificity, miRNA-25—which has the same length (21 bases) as miRNA-16b and coexists with miRNA-16b in pregnant cows—was tested as the interference, using the sensors with the same DNA probe. The results showed no observable response and confirmed the specificity of the miRNA sensor.

## 2. Materials and Methods

### 2.1. Interfacial Capacitance Sensing

**Figure 1:**
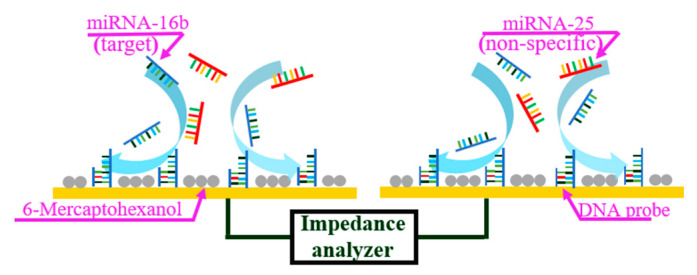
ACET microflow accelerates the binding of miRNA-16b and DNA probe.

The miRNA sensor in this study was based on ACEK capacitive sensing, and used to detect the binding of miRNA-16b molecules with the immobilized DNA probes. The single-stranded DNA probes were immobilized on the interdigitated microelectrodes, which captured and bound with the miRNA-16b molecules. The ssDNA (with the sequence of 5′- GCCAATATTTACGTGCTGCTGCTA-3′) and the miRNA-16b (with the sequence of UAGCAGCACGÂUAAAUAUUGGC) were complementary strands. MiRNA-25 (with the sequence of CAUUGCACUUGUCUCGGUCUG), which has the same length (21 bases) as miRNA-16b and coexists with it in pregnant cows, was set as negative control. Open sites on the surface were then blocked with 6-mercaptohexanol (6-MH). During detection, the microelectrodes were stimulated with a low voltage (300 mVrms) AC signal to induce ACEK effects, such as the AC electrothermal (ACET) effect, to route the miRNA-16b molecules around the electrodes onto the electrode surface and hybridize with the immobilized DNA probes. The binding between miRNA-16b molecules and the immobilized DNA probes led to a change of the interfacial capacitance (Cint) at the microelectrode surface, which was detected electrically using the same AC signal, as shown in Figure 1.

**Figure 2:**
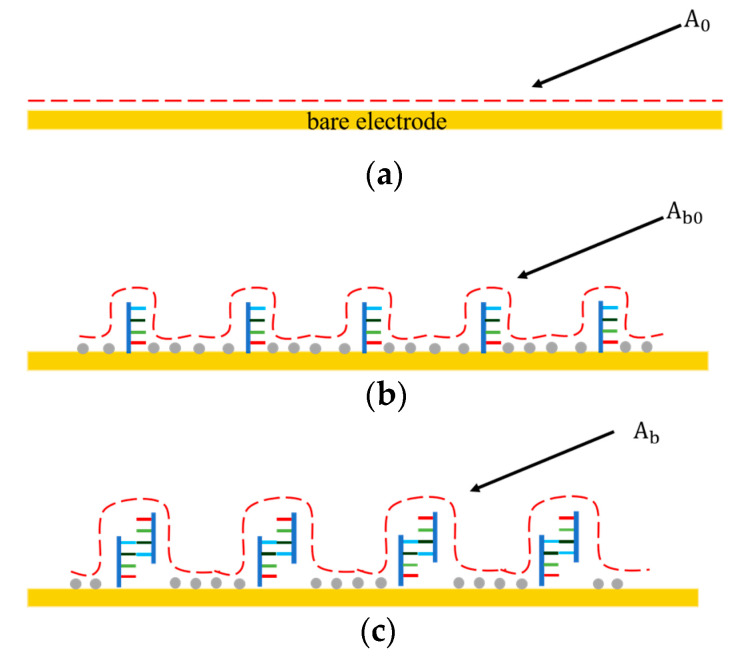
The interfacial capacitance changes at different stages of probe immobilization and miRNA-16 binding. (**a**) Before probe immobilization. (**b**) After probe immobilization and blocking. (**c**) After miRNA-16b binding with DNA probe.

As shown in Figure 2, before the DNA probe was immobilized on the electrode surface, the interfacial capacitance was determined by the formation of electrical double layer (EDL) (Figure 2a). $A_{0}$ represented the surface area of the interfacial capacitance before immobilization. After functionalization, the electrode surface was covered with the DNA probes and 6-MH at high density. Such a DNA/6-MH monolayer could be modeled as a dielectric capacitance that serially connects with the initial EDL capacitance (Figure 2b), while $A_{b0}$ represents the surface area of the interfacial capacitance after immobilization of the probe DNA and 6-MH. After miRNA-16b targets bind with the probe DNA (Figure 2c), the total interfacial capacitance changes again, where $A_{b}$ represents the surface area of the interfacial capacitance after binding. The capacitance change predominantly results from an increase in the surface area of the interfacial capacitance, which can be used for the detection of miRNA-16b.

**Figure 3:**
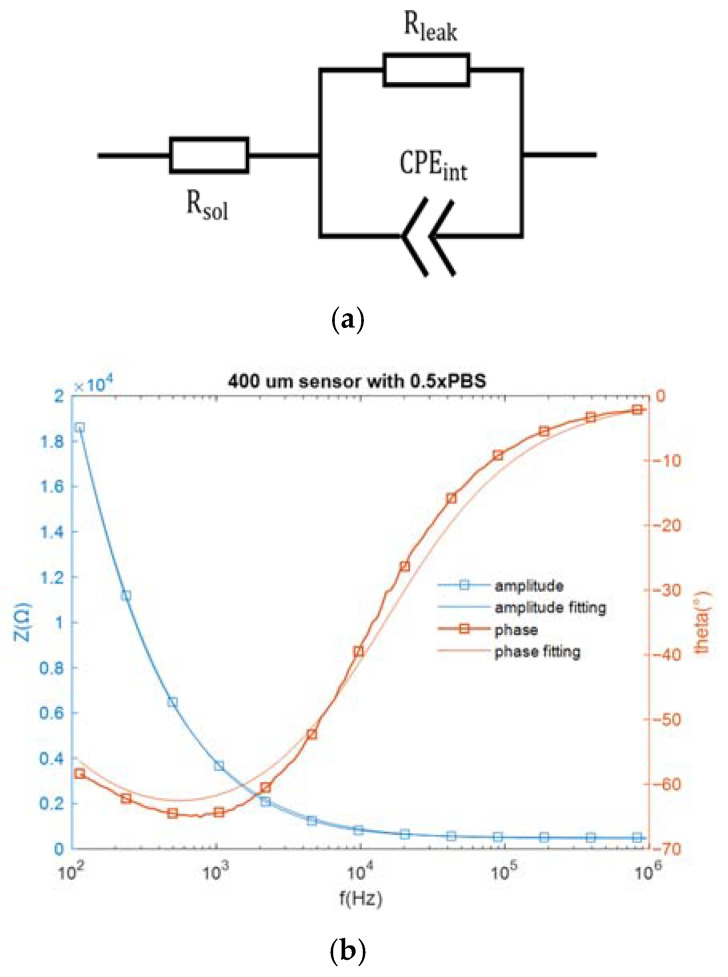
(**a**) The equivalent circuit network for an electrode biosensor, and (**b**) the measured impedance spectra and the fitting spectra.

As mentioned above, the sensor in this work could be represented by an equivalent electrical network. Specifically, when the interdigitated electrodes are immersed in an electrolytic solution, the electrode cell can be represented by an equivalent circuit network consisting of a solution resistance ($R_{\mathrm{sol}})$, an interfacial capacitance $\left(C_{\mathrm{int}}\right)$ modeled by a constant phase element $\left({\mathrm{CPE}}_{\mathrm{int}}\right)$ and a resistive path $\left(R_{\mathrm{leak}}\right)\;$in parallel with this capacitance for the non-faradaic sensors [22]. The complex impedance of a CPE can be expressed by 1/${\left(j\omega A\right)}^{m}$, where A is analogous to a capacitance, ω is the frequency in rad/s, and 0.5 < m < 1 (m = 1 corresponds to a capacitor and m = 0.5 corresponds to a Warburg element) [19]. The bare sensor impedance was measured over a frequency range of 100 Hz to 1 MHz, with a buffer conductivity equivalent to that of 1× Saline Sodium Citrate buffer (SSC) placed over the IDE. The equivalent circuit network and the impedance spectra are both shown in Figure 3.

The component values in the equivalent circuit network can be estimated from the fitting data. The solution resistance $R_{\mathrm{sol}}$ is 454.4 Ω and the resistive path $R_{\mathrm{leak}}$ equals 7.73 × 10^−4^ Ω. As for the constant phase element ${\mathrm{CPE}}_{\mathrm{int}}$, which can be expressed by $1/{\left(j\omega A\right)}^{m}$, A = 4.465 × $10^{-9}$ and m = 0.7842. At the assay frequencies of 10 kHz and 100 kHz, ${\mathrm{CPE}}_{\mathrm{int}}$ equals 2578.8 Ω and 423.85 Ω, respectively, which are much smaller than $R_{\mathrm{leak}}$. Since electricity takes the path of least resistance, the impedance network can be simplified as a series connection of the constant phase element $\left({\mathrm{CPE}}_{\mathrm{int}}\right)$ and the solution resistance ($R_{\mathrm{sol}})$. In this work, the miRNA-16b detection was based on non-faradaic impedance measurement, and the interfacial capacitance $C_{\mathrm{int}}$, which closely correlates with constant phase element $\left({\mathrm{CPE}}_{\mathrm{int}}\right)$, was monitored for target detection.

### 2.2. ACEK Enrichment Mechanism

In the process of detection, target molecules tended to diffuse to the electrode surface and bind with the immobilized probes. The random nature of passive diffusion is the underlying cause for low sensitivity and long testing times. To overcome these limitations for detection, ACEK effects were applied for molecule enrichment. Specifically, ACEK effects include dielectrophoresis (DEP) [23], AC electroosmosis (ACEO) [24] and AC electrothermal (ACET) effect [25]. For ACEO, the flow velocity diminished to close to zero when the conductivity of solution was increased to above 0.085 S/m [26]. Since the miRNA-16b sample solutions were based on 0.5× SSC buffer and 1× SSC buffer, with conductivities of 0.433 S/m and 0.865 S/m, ACEO flows were negligible in this work. In order to stimulate the fluids with high conductivity to convect to the electrode surface, the ACET effect was applied as we reported earlier [26]. ACET effects applied volume force on fluid and the microflows were generated to accelerate miRNA-16b molecules traveling to the surface of the electrode for binding, as shown in Figure 1. The high efficiency of binding shortened the testing time to 30 s and enhanced the sensitivity for detection [26]. The DEP force and ACET force can be expressed as

(1) FDEP=2πa3εmRe[εp*−εm*εp*+2εm*]∇|E|2

(2) $$F_{\mathrm{ACET}}=-0.011\nabla {T\varepsilon }_{m}{\left|E\right|}^{2}$$

where a is the particle radius, $\varepsilon_{m}\;$is the medium permittivity,|E| is electric field modulus, $\varepsilon_{p}^{*}$ and $\varepsilon_{m}^{*}$ are complex permittivities of particle and medium and ∇T is temperature gradient. As DEP force scales with particle size while the ACET is size-independent [25], the ACET effect was expected to be more effective than DEP for miRNA-16b enrichment due to miRNA-16b’s small size [25,26]. The detailed analysis of the miRNA-16b enrichment mechanism is exhibited in Section 3.2, based on the experiment results.

### 2.3. Sensor Fabrication

Single-sided highly flexible clad laminate (PulsarProFX^®^) was used for the device prototyping. The laminate was a 0.005″ thick FR4 fiberglass with 1/2 oz copper. Interdigitated electrodes were patterned by PCB technique. The PCB fabrication process is simple and can be done in just a few minutes using benchtop equipment. The detailed sensor fabrication steps were as follows: (1) Draw the electrode pattern in Microsoft Visio and then print it onto a piece of toner transfer paper (PulsarProFX) using a regular laser printer. (2) Cover the copper laminate with the patterned toner transfer paper and roll it through a laminator (Micro-Mark, model VL110) when the ‘HOT’ indicator is on. Soak the copper laminate together with the toner transfer paper in water for several seconds and remove the toner transfer paper. The electrode pattern is then transferred to the copper laminate. (3) Cover the copper laminate with a piece of toner reactive foil (TRF) and roll it through the laminator. After removing the TRF from the copper laminate, only the electrode pattern on the copper laminate was covered by TRF, which is etch-resistant. (4) Heat 30 mL of ferric chloride solution (MG Chemicals) to 55 °C and put the copper laminate into the solution. Copper was etched within 10 min and the PCB showed the desired electrode pattern. Rinse the PCB with acetone and isopropyl alcohol to remove TRF from the electrode surface. Clean the PCB under running de-ionized water for 5 min. (5) Solder two lead wires to the electrode pads. (6) Smoothen the electrode surface with sandpaper (#5000 Grit) and clean it thoroughly. (7) Finally, coat the electrode with a thin layer of gold by electroplating. The plating solution used here was Caswell^®^ 24CT Gold Plating Solution. An image of the fabricated interdigitated electrodes is provided in Figure S1 in Supplementary Materials. The characteristic length of the sensor was 400 µm (with widths of 400 µm and 400 µm gaps). The adhesive chamber in Figure S1b is Press-to-Seal^®^ silicon isolator with a 2.5 mm diameter and a 2 mm depth.

### 2.4. Sample Preparation

Single-strained DNA probe for miRNA-16b target recognition was purchased from Integrated DNA Technologies, Inc. (IDT, Coralville, IA). Its sequence was 5′- GCCAATATTTACGTGCTGCTGCTA-3′ while the 5′-end was dithiol-modified for self-assembly on the gold surface. Before experiments, the ssDNA probes were further reduced using TCEP (Sigma Aldrich, USA). The miRNA-16b was purchased from IDT and its sequence was rUrArG rCrArG rCrArC rGrUrA rArArU rArUrU rGrGrC [10]. For the sensor selectivity test, miRNA-25 [11], also from IDT, had the following sequence: rCrArU rUrGrC rArCrU rUrGrU rCrUrC rGrGrU rCrUrG. After DNA probe incubation, the surface of the electrode was blocked with 1 mM 6-MH (Sigma-Aldrich, St. Louis, MO, USA). For probe solution, ultrapure water (Mili-Q) and 0.05× PBS (Thermo Fisher Scientific, Waltham, MA, USA) were used to prepare 20 µM DNA probe solution. For testing, miRNA-16 was diluted in 1× SSC buffer (AccuGENE™, Lonza, Rockland, ME) to make stock solutions. The final concentrations of miRNA-16b samples for testing were: 100 fM, 10 fM, 1 fM and 0.1 fM.

### 2.5. Preparation of Microelectrode Chips

Before immobilizing the DNA probe on the electrode, the electrodes needed to be cleaned rigorously. The steps for electrode cleaning are reported in our earlier work [27]. Briefly, the electrode chips were cleaned with acetone, isopropyl alcohol (IPA), ultrapure water, and then air-dried. After these steps, electrode chips were transferred into a UV ozone cleaner for 15 min. Then, the electrode chips were ready for probe functionalization.

The 10 µL DNA probe solution (100 µM in 0.05× PBS) was loaded on the electrode surface for incubation and the electrode chips were kept in humidor for 18 h. Thereafter, electrode chips needed to be blocked with 1 mM 6-MH for 3 h. Finally, the electrode chips were ready for detection.

### 2.6. Measurement and Analysis

As mentioned in Section 2.1, capacitance values were continuously measured to calculate the capacitance change ratio. In this work, the interfacial capacitance was measured for 30 s and sampled at 120 points with an AC signal of 10 kHz and 300 mV. The capacitance change ratio is expressed as normalized capacitance versus time (%/min), calculated by the least square linear fitting. The concentration of target samples from 0.1 fM to 100 fM were tested five times using a new functionalized sensor each time. An impedance analyzer was used for data acquisition (Aglient 4294A).

Before testing, the miRNA samples needed to be heated in a water bath at 95 °C for 5 min and then cooled on ice for 5 min. To obtain the best performance of the miRNA-16b detection sensor, the frequency was optimized and the sensors were prepared with different incubation buffers and testing buffers. All the target samples (miRNA-16b and miRNA-25) at different concentrations were tested five times and the average values were used for analysis.

## 3. Results

### 3.1. Treatment for miRNA-16b Samples

We used one single loop of heating and fast cooling to linearize miRNA for effective binding to the probe DNA. The first experiment was to confirm whether heat and fast annealing led to improvement, before other optimizations were employed. The melting temperature (Tm) of miRNAs are around 45 °C, so the samples were heated to 95 °C then put on ice to cool. We compared the responses of miRNA-16b samples that were not heated and cooled with the miRNA-16b samples that were heated in the water bath at 95 °C (5 min) and cooled on ice (1 min). The results are provided in Figure S2 in Supplementary Materials. The miRNA-16b samples were at a fixed concentration of 1 fM. The response of temperature cycled samples was 3.117%/min, while the response for the untreated sample was 2.677%/min. The 16.44% response improvement proved that temperature treatment for miRNA-16b samples is necessary. Therefore, in all the following experiments, all miRNA samples were heated and ice-cooled.

### 3.2. Frequency Optimization

**Figure 4:**
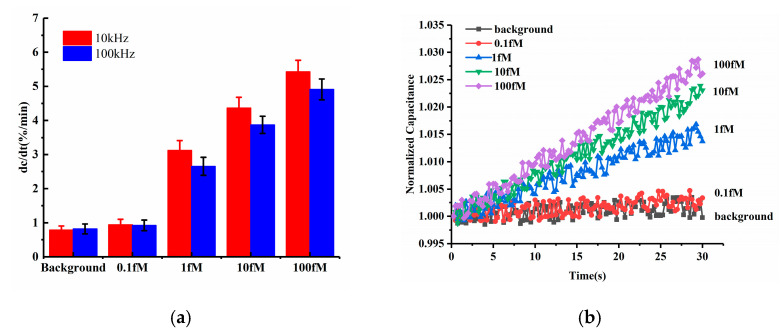
(**a**) Detection results at 100 kHz and 10 kHz (**b**) Normalized capacitance change as a function of time within 30 s for various levels of miRNA-16b.

Among ACEK effects, the ACET effect is not frequency dependent, while DEP forces may strongly depend on the applied frequency. Both of these ACEK forces may occur when an AC signal is applied. Thus, it was necessary to test different frequencies of the measuring signal, in order to determine the dominant ACEK mechanism. Therefore, two frequencies of 10 kHz and 100 kHz were used in the comparison experiments for the detection of miRNA-16b. The testing results are shown in Figure 4a. At the frequency of 10 kHz, the sensor responses of the background solution and 0.1 fM miRNA-16b were 0.788%/min and 0.943%/min, respectively, while the responses were 0.821%/min and 0.924%/min, respectively, when using 100 kHz. There were no significant differences between them. Also, under the frequency of 10 kHz, the 1 fM, 10 fM and 100 fM miRNA-16b showed responses of 3.117%/min, 4.366%/min and 5.428%/min, respectively. As for 100 kHz, the responses of 1 fM, 10 fM and 100 fM miRNA-16b decreased to 2.653%/min, 3.873%/min and 4.912%/min, respectively, and the differences in responses were about −14.89%, −11.29% and −9.51%, respectively, when compared with the cases at 10 kHz. These differences (~10%) indicated that the response of the sensor was relatively insensitive to the measuring frequency, pointing to the ACET effect’s dominant sensing over DEP [28].

The representative curves of normalized capacitances for different miRNA-16b concentrations are displayed in Figure 4b, corresponding to the 10 kHz data in Figure 3a. The capacitances were shown to linearly increase with time, and the capacitance change rates were commensurate with increasing concentrations of miRNA-16b, due to increasing levels of miRNA-16b binding.

Furthermore, according to the equivalent circuit network and the electrical impedance spectra discussed in Section 2.1, the impedance network can be simplified as a series connection of the constant phase element $\left({\mathrm{CPE}}_{\mathrm{int}}\right)$ and the solution resistance ($R_{\mathrm{sol}})$. Compared to applying 100 kHz, when using 10 kHz, the ${\mathrm{CPE}}_{\mathrm{int}}$ showed more capacitive components and resulted in a greater response of the capacitive sensor. Therefore, for frequency optimization, 10 kHz was selected to perform the tests.

Previous work demonstrated that the detection of lipopolysaccharides and genomic DNA was more pronounced at 100 kHz [29,30]. Because both were larger than the miRNA-16b tested here, the DEP effect was much more pronounced for enrichment. Thereby, it was reasonable to have a better response at 10 kHz for miRNA-16b detection due to the dominance of the ACET effect for miRNA enrichment [24,31].

**Figure 5:**
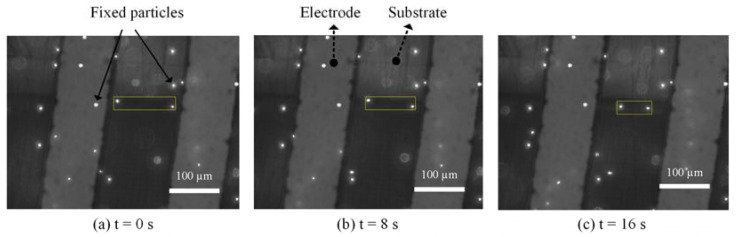
Image series showing particle movement in 0.5× SSC at 10 kHz.

To directly verify that the main effect of AC signal under our test conditions was ACET, we performed a microfluidic experiment based on fluorescent tracing particles. The solution of fluorescent particles (FluoSpheres^®^ Carboxylate-Modified Microspheres, 1 µm, nile red fluorescent 535/575, 2% solids) was diluted 1000 times by 0.5× SSC. Even though both ACEO and ACET microflows were generated by nonuniform electric fields between electrodes, for electrodes with a thermally insulative substrate, flow patterns were fundamentally different [32,33]. While ACEO flows went from the electrode edge to its inside, ACET flows followed the direction of thermal gradients, i.e., going opposite to the ACEO directions, in this case. Thus, at the surface of side-by-side PCB electrodes, ACET flows went from electrode to gap, whereas ACEO flowed from gap to electrode. The particle movements at the surface of PCB electrodes under 10 kHz-7 Vpp signals are shown in Figure 5. Two moving particles are boxed out with a yellow box, indicating their distances at 0, 8 and 16 s. It shows that the two particles were moving away from the electrodes toward the gap, verifying that the particles were carried by ACET microflows.

### 3.3. Optimization of Sensor Preparation

**Figure 6:**
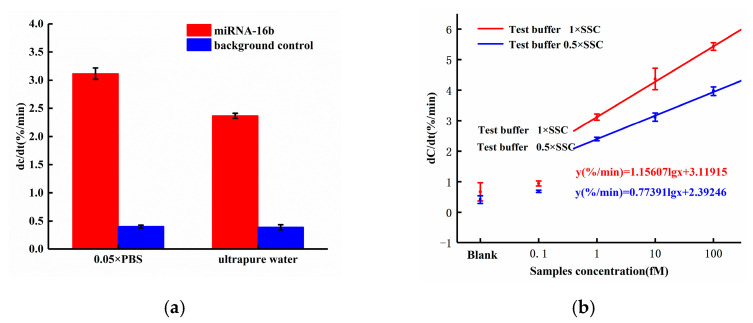
(**a**) Sensor response with probe diluting in 0.05× PBS and ultrapure water; (**b**) Dose responses with miRNA-16b diluting in 0.5× SSC and 1× SSC.

Based on our earlier work, the ACEK-enhanced biosensor for biomolecule detection was expected to have different performance on responses, when different probe buffers or target buffers were used [18]. In this work, we compared the sensor responses using 0.05× PBS and ultrapure water for probe immobilization, respectively, and miRNA-16b samples with a fixed concentration of 1 fM were tested. The sensor responses are shown in Figure 6a. When applying 0.05× PBS solution to dissolve DNA probes, the response of miRNA-16b with the concentration of 1fM was 3.117%/min, which was up to 31.69% higher than the response (2.367%/min) with ultrapure water dissolving the DNA probe. This was because there were more ions in the 0.05× PBS solution than in ultrapure water, and the ions in the solution shielded the charge of nucleic acid. Thus, when the DNA probes were attached to the electrode surface, the coverage area increased and resulted in the improvement of binding efficiency.

To optimize the concentration of the target buffer, we compared the results of diluting miRNA-16b molecules with 0.5× SSC solution and 1× SSC solution. The sensor responses are shown in Figure 6b.

According to Figure 6b, when the miRNA-16b molecule was diluted with 0.5× SSC solution, the responses of the miRNA-16b sample with concentrations of 1fM, 10fM, and 100 fM were 2.397%/min, 3.117%/min, and 3.967%/min, respectively. When the miRNA-16b molecule was diluted with 1× SSC solution, the responses of the miRNA-16b samples with concentrations of 1fM, 10fM, and 100fM were 3.117%/min, 4.366%/min and 5.428%/min, respectively. Compared with diluting targets using 0.5× SSC, miRNA-16b molecules diluted with 1× SSC solution increased the sensor response by 30.03%, 40.07% and 36.83%, respectively, with target concentrations of 1 fM, 10 fM, and 100 fM. In this work, the ACET effect accelerated the enrichment of miRNA-16b molecules at the electrode surface. Compared with the 0.5 × SSC solution, the conductivity of the 1 × SSC solution was higher and would generate stronger ACET microflows for target enrichment. Thus, the efficiency of binding between the DNA probe and miRNA-16b were improved, and the desired enhancement of the sensor response was achieved.

### 3.4. Dose Response of miRNA-16b Detection

To obtain peak performance of our sensor, we chose 0.05× PBS solution (5 mM phosphate buffer [pH 7] containing 7.5 mM sodium chloride) to dilute the DNA probe for incubation and 1× SSC solution as the background solution for the miRNA-16b molecules. The concentration of target samples from 0.1 fM to 100 fM were tested five times, and the average values were used to analyze specific responses to various concentrations of miRNA-16b.

**Figure 7:**
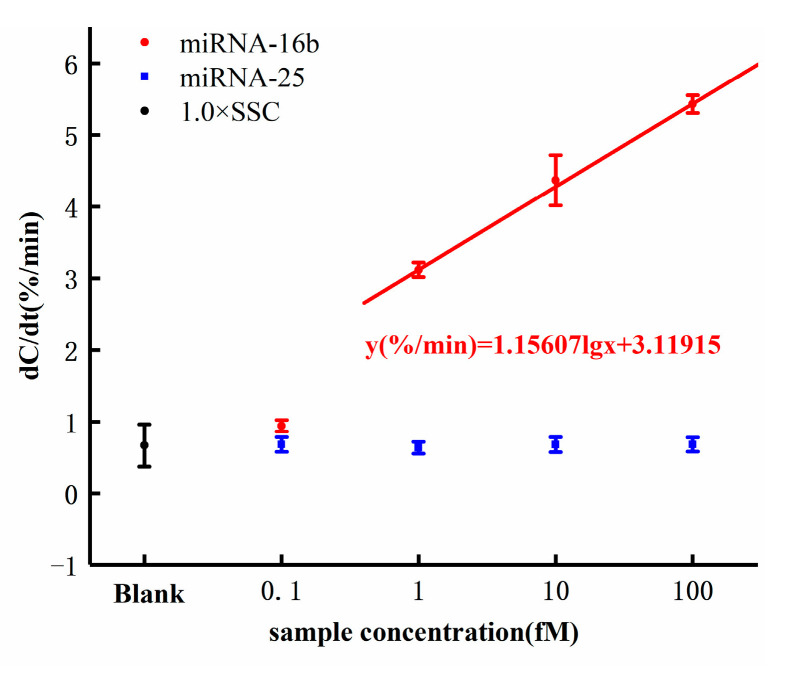
Capacitance change rates in response to different concentrations of miRNA-16b and miRNA-25 concentrations.

The dose response of miRNA-16b detection is shown in Figure 7. As shown in Figure 7, the response of background solution 1× SSC was 0.788%/min, which was less than 1%/min that of the miRNA-16b samples. The significant response difference proved that the increase of interfacial capacitance resulting from the hybridization of miRNA-16b and DNA probe and the background solution of the samples did not bring any interference to the detection and did not cause the capacitance change. The response of the miRNA-16b sample with concentration of 0.1 fM was 0.943%/min. The response was less than 1%/min, indicating that the sensor detection limit was higher than 0.1 fM. The response of the miRNA-16b sample at 1 fM was 3.117%/min, that at 10 fM was 4.366%/min, and that at 100 fM was 5.428%/min. All the response values were greater than the background response, indicating that the electrode surface interface capacitance increased during the hybridization of the DNA probe and miRNA-16b.

According to the equations introduced in Section 2.1, the increase of capacitance value indicated that additional topological structure was introduced when the hybridization occurred between miRNA-16b and DNA probe, resulting in increased surface area and leading to increased interface capacitance. More importantly, the response increased with the increase of the concentration of miRNA-16b samples following a semilogarithmic relationship. The dose response of the sensor can be expressed by the linear fitting equation y(%/min) = 1.15607 lg(x) + 3.11915, where x is the concentration of the miRNA-16b analytes with the unit of femtomolar. The cut-off response of the sensor was defined as three standard deviations from the response of the background solution. By inserting the cut-off response (1.676%/min) into the dose response equation y(%/min) = 1.15607 lg(x) + 3.11915, the detection limit of the sensor can be calculated to be 0.056 fM (theoretical value). However, this value (0.056 fM) was less than the lowest concentration tested (0.1 fM), from which we did not obtain a good response. Therefore, the detection limit of the sensor is theoretical.

### 3.5. Selectivity

MiRNA-25 was used as a negative control to evaluate the selectivity of the biosensor we designed. As shown in Figure 7, the responses of miRNA-25 at the concentrations of 0.1 fM, 1 fM, 10 fM, and 100 fM were less than 1%/min, and the responses were independent of the concentration. This showed that the selected DNA probe specifically hybridized with the target miRNA-16b but not with the irrelevant RNA, miRNA-25.

### 3.6. Spiked Serum Samples Detetction

To further validate whether this miRNA sensor could be applied to test complex matrix, miRNA-16b spiked bovine serum samples were also tested as a prove of concept. First, negative serum samples were mixed as negative serum pool, which was further 1:10 diluted using 0.5× PBS. Then, miRNA-16b samples (ranging from 0.1 fM to 100 fM) were spiked into diluted serum pool to simulate positive serum samples. To test the selectivity, the spiked miRNA-25 was also tested as a negative control.

**Figure 8:**
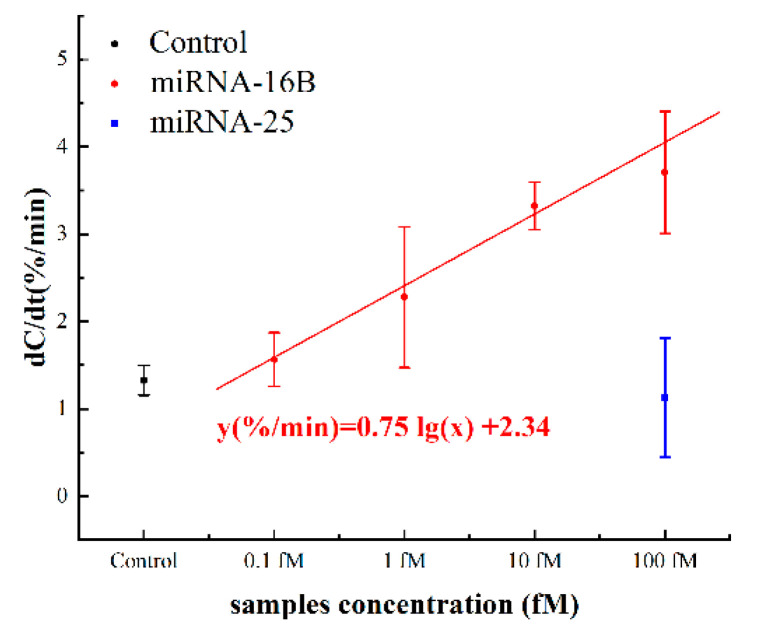
Capacitance change rates in response to different concentrations of spiked miRNA-16b and miRNA-25 serum samples.

The capacitance change rates are shown in Figure 8. The normalized capacitance changes for 0.1 fM to 100 fM were 1.536 ± 0.307%/min, 2.276 ± 0.806%/min, 3.323 ± 0.272%/min and 3.706 ± 0.698%/min, respectively. The response of control background (negative serum pool) was 1.327 ± 0.171%/min, which was slightly below the response of spiked 0.1 fM miRNA. The dose response can be represented by the linear fitting equation y(%/min) = 0.75 lg(x) + 2.34, where x is the concentration of the miRNA-16b analytes in fM. The cut-off response of sensor was defined as three standard deviations (1.840%/min) from the response of background solution, which was calculated to be 0.215 fM. Considering the dilution, the detection limit was 4.30 fM in neat serum, theoretically. Furthermore, the responses of the 1 fM samples and the control were statistically examined by *t*-test and the *p*-value was 0.03. This statistic result indicated that, with 5% serum samples, the signal-noise ratio of our sensor was sufficiently high for the detection of >1 fM miRNA from complex metrices. The results of spiked serum tests further supported that the sensor could be applied to real serum samples. As miRNA-16b at 1 fM or above in serum has already been proven to be a biomarker used to diagnose successful bovine pregnancy 8 days earlier than current tests [21], the miRNA detection method described in this study will help dairy farmers to avoid unnecessary animal management costs during that time period.

## 4. Conclusions

This work presented a new method for detection of miRNA using ACEK-enhanced capacitive sensing. The ACET effect was utilized to accelerate the transport of miRNA and helped to accomplish the rapid and sensitive detection of miRNA within 30 s. The detection limit of the sensor was 1 fM in SSC or diluted serum. This work may form a basis for the development of point-of-care miRNA detection in general. Since the model miRNA used in this study, miRNA-16b, was shown to be a biomarker for early pregnancy in dairy cows, a specific application would be to realize a rapid, sensitive, simple, and inexpensive diagnostic for early bovine pregnancy detection. Further study would include testing more miRNA molecules for specificity—and clinical samples with qPCR confirmation.

## Data Availability

The data used to support the findings of this study are available from the corresponding authors upon request.

## Supplementary Materials

The following are available online at https://www.mdpi.com/article/10.3390/s21123985/s1, Figure S1: The interdigitated electrode. (a) bare electrode. (b) electrode with silicon chamber, Figure S2, Responses of miRNA-16b samples through water bath treatment and without water bath treatment.
