# Supplementary material for: Rapid and Sensitive Detection of miRNA Based on AC Electrokinetic Capacitive Sensing for Point-of-Care Applications
Source: Sensors (Basel). 2021 Jun 9;21(12):3985. doi: 10.3390/s21123985 (PMC8226656; doi:10.3390/s21123985)
Supplement: Supplementary file 1 [file sensors-21-03985-s001.zip › sensors-1179547-supplementary.pdf]

Rapid and sensitive detection of miRNA based on AC electrokinetic capacitive sensing for point of care applications

Supplementary materials

1. The fabricated interdigitated electrode including bare electrode and electrode with silicon chamber.

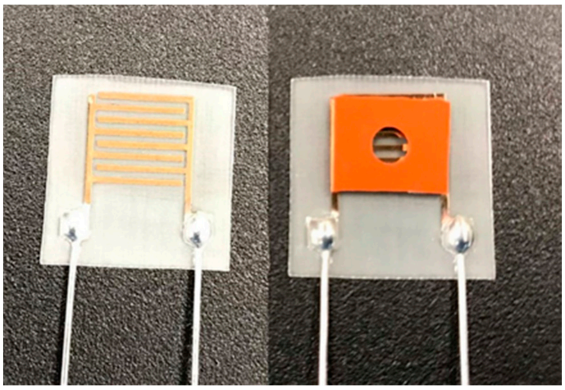

(a) (b)

Figure S1. The interdigitated electrode. (a) bare electrode. (b) electrode with silicon chamber.

2. The Responses of miRNA-16b samples through water bath treatment and without water bath treatment.

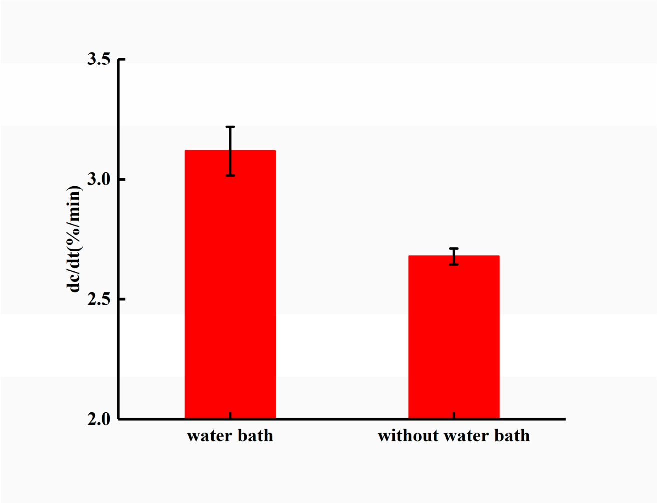

Figure S2. Responses of miRNA-16b samples through water bath treatment and without water bath treatment.
